# ClinQC: a tool for quality control and cleaning of Sanger and NGS data in clinical research

**DOI:** 10.1186/s12859-016-0915-y

**Published:** 2016-02-02

**Authors:** Ram Vinay Pandey, Stephan Pabinger, Albert Kriegner, Andreas Weinhäusel

**Affiliations:** Health & Environment Department, Molecular Diagnostics, AIT Austrian Institute of Technology GmbH, Vienna, Austria; Institut für Populationsgenetik, Vetmeduni Vienna, Veterinärplatz 1, A-1210 Vienna, Austria

**Keywords:** Sanger sequencing, Next generation sequencing, Quality control, Molecular diagnostic testing

## Abstract

**Background:**

Traditional Sanger sequencing has been used as a gold standard method for genetic testing in clinic to perform single gene test, which has been a cumbersome and expensive method to test several genes in heterogeneous disease such as cancer. With the advent of Next Generation Sequencing technologies, which produce data on unprecedented speed in a cost effective manner have overcome the limitation of Sanger sequencing. Therefore, for the efficient and affordable genetic testing, Next Generation Sequencing has been used as a complementary method with Sanger sequencing for disease causing mutation identification and confirmation in clinical research. However, in order to identify the potential disease causing mutations with great sensitivity and specificity it is essential to ensure high quality sequencing data. Therefore, integrated software tools are lacking which can analyze Sanger and NGS data together and eliminate platform specific sequencing errors, low quality reads and support the analysis of several sample/patients data set in a single run.

**Results:**

We have developed ClinQC, a flexible and user-friendly pipeline for format conversion, quality control, trimming and filtering of raw sequencing data generated from Sanger sequencing and three NGS sequencing platforms including Illumina, 454 and Ion Torrent. First, ClinQC convert input read files from their native formats to a common FASTQ format and remove adapters, and PCR primers. Next, it split bar-coded samples, filter duplicates, contamination and low quality sequences and generates a QC report. ClinQC output high quality reads in FASTQ format with Sanger quality encoding, which can be directly used in down-stream analysis. It can analyze hundreds of sample/patients data in a single run and generate unified output files for both Sanger and NGS sequencing data. Our tool is expected to be very useful for quality control and format conversion of Sanger and NGS data to facilitate improved downstream analysis and mutation screening.

**Conclusions:**

ClinQC is a powerful and easy to handle pipeline for quality control and trimming in clinical research. ClinQC is written in Python with multiprocessing capability, run on all major operating systems and is available at https://sourceforge.net/projects/clinqc.

**Electronic supplementary material:**

The online version of this article (doi:10.1186/s12859-016-0915-y) contains supplementary material, which is available to authorized users.

## Background

Due to the rapid growth in sequencing throughput, cost reduction, improved sequencing chemistry, and the possibility to multiplex several sample/patients in a single sequencing experiment, Next Generation Sequencing (NGS) has become a powerful and efficient tool for disease causing variant identification and decoding of a number of genetically heterogeneous diseases including cancer [1]. While NGS technologies have been used to identify variants in several patients in a cost and time effective manner, Sanger sequencing has been used as a complementary method to narrow down and confirm the NGS-detected variants before making clinical decision [2–4]. In order to identify the potential disease causing mutations with great accuracy, it is essential to use only high quality reads. Therefore, integrated software tools are required, which can eliminate platform specific sequencing errors as well as low quality reads, and perform format conversion, quality trimming and filtering.

Moreover, they should be able to analyze several sample/patients data generated from both Sanger and NGS platforms in a single run and provide execution flexibility by using requirement based customized parameters [5].

At present, several solutions are available for NGS data quality control such as NGS QC Toolkit [6], FastQC [7], PRINSEQ [8], TagDust [9], FASTX-Toolkit [10], SolexaQA [11], TagCleaner [12], CANGS [13], ngs_backbone [14], Galaxy [15], SIMPLEX [16] and QC-Chain [17]. Many of these tools work only for a particular NGS sequencing platform, are limited in their functionality (such as specific input format requirements) and none supports Sanger sequencing format conversion, quality control, trimming and base calling. Therefore, a one-stop, integrated and easy-to-use software tool to analyze Sanger as well as NGS sequencing data is needed, which offers easy handling of input and output data and support analysis of multiple sample/patients in a single run.

We have developed ClinQC, a flexible, integrated and easy-to-use solution for sequencing data processing, format conversion and quality control for Sanger and three NGS platforms including Illumina, 454 and Ion Torrent. We anticipate that this tool will be extremely useful for initial file processing, quality control and format conversion in sequencing based clinical and genomic research studies for expert and not-expert users.

## Implementation

**Fig. 1:**
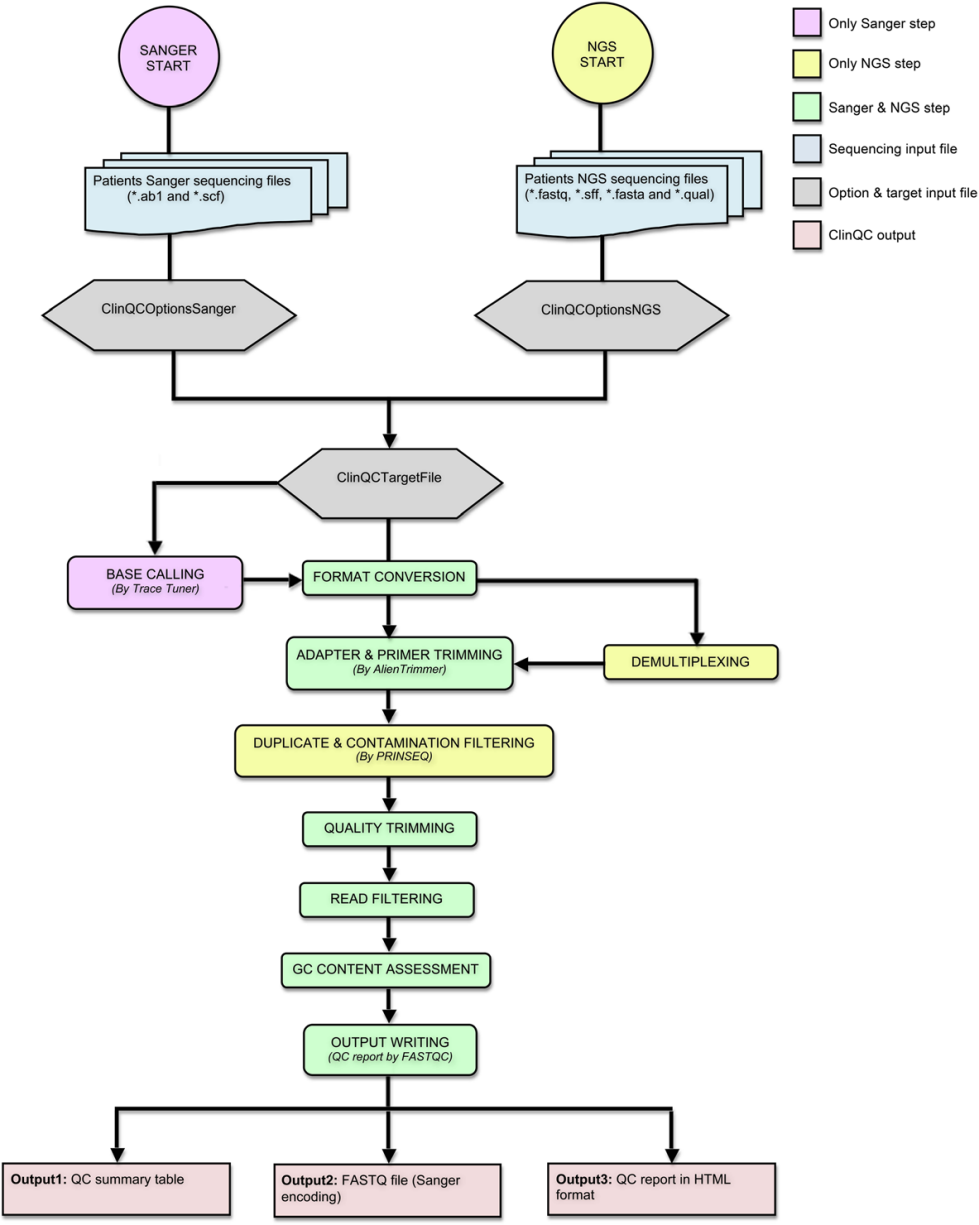
The workflow of ClinQC pipeline. ClinQC tool can be run with a single command. The flow of analysis is depicted from top to bottom. BASE CALLING (*violet color*) step is only applicable for Sanger data analysis; DEMULTIPLEXING and DUPLICATE & CONATMINATION FILTERING (*yellow color*) steps are only applicable for NGS data analysis; all other steps (*green color*) are applicable for both analysis flows. ClinQC generates three final outputs

ClinQC tool is developed in Python 2.7.9 (http://www.python.org) by using the multiprocessing capability. It uses four other tools including FASTQC [7], PRINSEQ [8], Alientrimmer [18], and TraceTuner [19]. The ClinQC workflow is depicted in Fig. 1 and consist of several sequential steps that lead from the raw sequencing reads to the high quality Sanger encoded FASTQ file for each patient/sample. All parameter settings can be specified in a single configuration file (Additional files 1 and 2). To achieve the optimized performance, ClinQC uses the available hardware (Physical memory and CPU) in a best possible way. A buffer file read write concept was implemented where input and output are partially stored in memory during the analysis, which reduces the computation time and reduces the disk reading and writing workload.

## Results and discussion

ClinQC is an open-source, easy-to-use and integrated tool, which facilitates the analysis of Sanger and NGS sequencing data in a single platform with a common input output model. It supports the rapid analysis of hundreds of sample/patient data in parallel. This pipeline provides full flexibility to customize all parameters using the “*ClinQCOptions”* file for handling the sequencing platform specific errors and provides proper guidelines for the analysis. All components of ClinQC workflow and their inputs have been summarized in Fig. 1.

### ClinQC pipeline

The ClinQC pipeline (Fig. 1) consists of nine sequential steps that starts with raw sequencing reads and ends up with three outputs: 1) QC summary table, 2) FASTQ files with high quality reads and 3) QC report. The detailed description of each step is given below:

1. Base calling

Due to unclear signal in Sanger pherogram files, the base caller of the sequencer always calls ambiguous nucleotide as N. However, it could output more specific ambiguous nucleotides, i.e., R, if signal is not clear between A or G; Y, if signal is not clear between C and T. Therefore, ClinQC uses the tool TraceTunner [19] to improve the base calling and assign more specific ambiguous nucleotides.

2. Format conversion

**Fig. 2:**
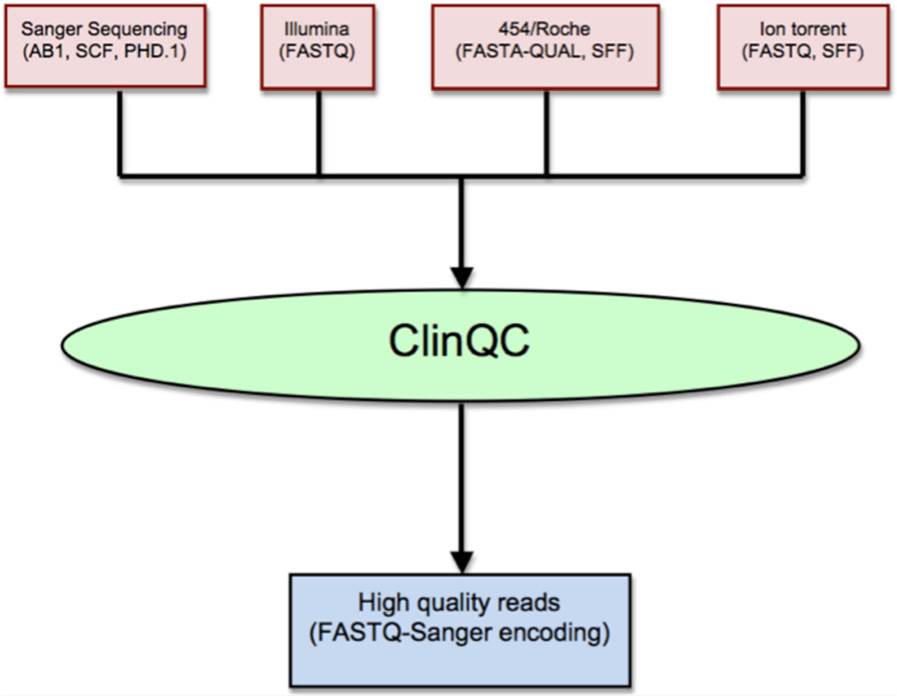
The format conversion workflow of ClinQC. ClinQC takes raw reads in any native file format of their sequencing platforms and returns a unified FASTQ files with Sanger (PHRED) quality encoding

In this step, ClinQC check the raw sequencing files and their formats and, if needed, converts from native file format to FASTQ with Sanger quality encoding (Fig. 2). Sanger sequencing files are accepted in AB1 and SCF format and NGS files are accepted in SFF, FASTA-QUAL and FASTQ format.

3. Demultiplexing

This step is only applicable for NGS data, where multiple samples are sequenced in a single sequencing run by using the multiplexing method. Based on the barcode sequences (MID: Multiplexed Identifier) provided in the *“ClinQCTargetFile”* file (as shown in Additional file 3), one FASTQ file per barcode is created. In case of paired-end sequencing, two FASTQ files (one for forward and one for reverse reads) are generated. This step will be skipped if the input data is already de-multiplexed.

4. Adapter and primer trimming

In this step, ClinQC trim the forward and reverse adapter and primer sequences provided in the *“PrimerAdapter”* file (as shown in Additional file 4) by using the AlienTrimmer [17] tool. AlienTrimmer is a flexible and sensitive sequence trimmer with mismatch tolerance, which allows the customization of the number of mismatches and k-mers based on the data quality and user requirements.

5. Duplicate and contamination filtering

PCR duplicates are a critical known problem, which arise when low abundant fragments are over amplified during the library preparation process. These duplicates can substantially inflate the allele frequency leading to wrong mutation detection and unexpected species richness in metagenomic analysis [20]. Therefore, ClinQC identify and remove duplicates using the PRINSEQ [8] tool to eliminate this technical artifact. Contamination is another problem particularly in metagenomic analysis [21] leading to wrong analysis when DNA from unknown sources is sequenced. Hence, ClinQC assesses and eliminates the contamination from the samples using the PRINSEQ [8] software.

6. Quality trimming

As NGS short read sequencing errors increase with the position in the read [22], ClinQC trim the low quality stretch and Ns from the 5’ and 3’ end of the reads.

7. Read filtering

In this Step ClinQC eliminate the reads, which do not meet the minimum average base quality and the minimum and maximum read length threshold. Thus, only high quality reads, which fulfill all quality trimming and filtering criteria, are kept in the final output file.

8. GC content assessment

GC content is crucial parameter when analyzing NGS data as the under or over representation of GC content could effect the downstream analysis and biological conclusions. Therefore, ClinQC reports the average GC content before and after QC in the summary table for each dataset.

9. Output generation

In this final step ClinQC write three output files: 1) summary output file in HTML format, 2) QC report, and 3) FASTQ files after filtering the low quality reads.

### ClinQC input

ClinQC provide a uniform input and output data models for Sanger and NGS sequencing data analysis requiring a minimum of three input files:

1. Target file: The target file contains experimental and sequencing information for each patient (Additional file 3). This file contains patient information including experiment details and raw sequencing files paths. The first column (Patient_ID) is mandatory and should be a unique identifier for each sample. Other patient information is optional and can be ignored for genomic data analysis.
2. Adapter-Primer file: This input file is optional and is required only if primer and adapter sequences need to be trimmed. It is a tab-separated text file with four columns describing the feature-type, id, forward sequence and reverse sequence (see Additional file 4).
3. ClinQCOptions file: The options file contains all input parameters for various parts of the pipeline and the path to the third party tools. A default ClinQCOptions file for Sanger and NGS data analysis is provided separately (Additional files 1 and 2).
4. Sequencing reads: ClinQC support Sanger sequencing reads in AB1 and SCF file format, Illumina reads in FASTQ format, 454 reads in SFF and FASTQ-QUAL format and Ion Torrent reads in SFF and FASTQ format.

### ClinQC output

**Fig. 3:**
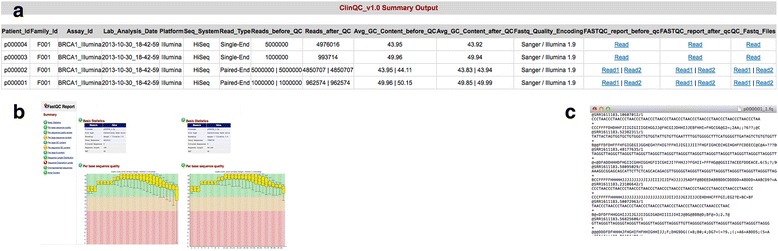
ClinQC final output. **a** QC summary table generated for each run, which includes experimental, patient, sequencing and QC information, one row for each sample/patient, (**b**) QC report generated by FASTQC before (*left*) and after (*right*) quality control for each sample/patient and linked in summary table, (**c**) FASTQ files with high quality reads for each sample/patient and linked in summary table

ClinQC produces output files in the same format for Sanger and NGS, which make output handling and further downstream analysis more efficient. The output files are:

1. QC summary table:

The QC summary table (Fig. 3a) consists of one line for each sample/patient including references to the two other patient specific output files (QC report and FASTQ file). The QC summary table contains experimental, patient, and sequencing information along with QC summary, number of reads and average GC content before and after quality control and filtering.

**Fig. 4:**
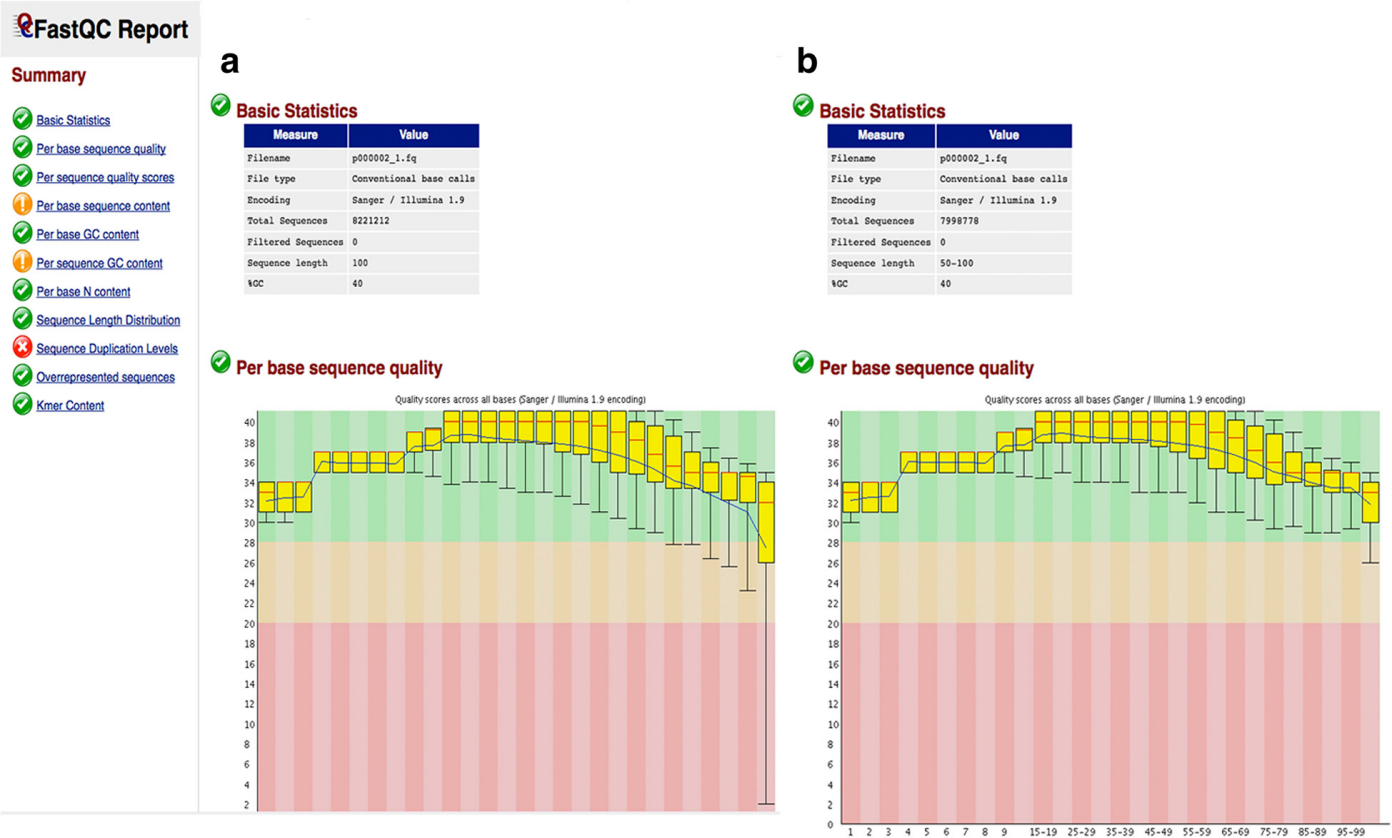
ClinQC quality control report generated by FASTQC. **a** Per base sequence quality before quality control and (**b**) per base sequence quality after quality control. ClinQC generates several useful QC plots for each patient’s FASTQ file before and after quality control. This feature enables to directly compare the data quality improvements and the number of filtered reads before and after quality control

2. QC report file:

After quality trimming and filtering, an extensive and intuitive quality report is generated in HTML format by using the widely used FASTQC [7] tool. It generates various useful plots (i.e. read base quality, read length distribution, overrepresented sequences and sequence duplication levels) to get a detailed view of the quality of sequencing data. ClinQC generates two QC reports for each patient/sample before QC (Fig. 4a) and after QC (Fig. 4b), which can be used for direct comparison. These two QC report HTML files are linked in the variant summary table.

3. FASTQ file with high quality reads:

After all file preprocessing, quality filtering and trimming steps are completed, ClinQC creates a Sanger encoded FASTQ file with high quality reads for each patient/sample (Fig. 2c). This file can be directly used in further down-stream analysis (e.g., mutation screening, genome assembly and metagenomic).

### Availability, installation and usage

ClinQC is an open-source pipeline and freely accessible for all researchers and clinicians. For non-expert users, ClinQC is available as a fully configured Virtual Machine (VM) accessible at https://sourceforge.net/p/clinqc/wiki/Virtual_Machine/, which is readily available and can be run on Virtual box (https://www.virtualbox.org/) without any installation and configuration requirement.

To use ClinQC outside of the Virtual Machine, we provide the source code of ClinQC along with pre-compiled third party tools/software separately for Linux and MacOS operating systems available from https://sourceforge.net/projects/clinqc/files/ClinQC_v1.0-linux.zip and https://sourceforge.net/projects/clinqc/files/ClinQC_v1.0-macos.zip respectively.

An extensive user manual (Additional file 5) is available from https://sourceforge.net/p/clinqc/wiki and a PDF version can be downloaded from https://sourceforge.net/projects/clinqc/files/ClinQC-Manual.pdf. The user manual includes description of the parameter file for NGS and Sanger, references to test datasets, dependency installation, ClinQC usage, and a detailed step-by-step description of the ClinQC pipeline. A test data set for Sanger, Illumina, 454 and Ion Torrent is available at https://sourceforge.net/projects/clinqc/files/test_data.zip

### Flexibility and reusability

ClinQC has been designed with focus on usability and organization of multiple sample/patient data with little manual task and user interaction for expert and non-expert users. Users just need to prepare one Target file (Additional file 3) and one option file (Additional files 1 and 2) for hundreds to thousands of sample/patient and are then able to run the whole pipeline with a single command. Since all input parameters and files are given in the Target file and the Option file, it would be extremely useful to preserve and store these files for each run for further use and reproducibility.

### An integrated pipeline

ClinQC is designed to cover a wide range of applications by supporting three NGS platforms as well as traditional Sanger sequencing trace files. In most clinical and genomic research studies, Sanger sequencing is being used in combination with a NGS sequencing platform for efficient and accurate mutation screening in a cost effective and time bound manner. Thus, providing Sanger and NGS data analysis under a unified single platform will help users with the analysis of sequencing data generated from one or more experiments.

The consistency of the paired-end relationship between forward and reverse read is essential to many subsequent analysis steps such as read mapping, variant calling or genome assembly. Therefore, ClinQC reads both read files simultaneously to maintain the paired-end consistency. In ClinQC, if one read of a pair is lost due to trimming or filtering, the corresponding pair is also excluded from the final data sets.

### Scalability

ClinQC is designed to handle sequencing data ranging from single-gene to whole genome sequencing. The software can be used to analyze several patient data in parallel from both Sanger and NGS sequencing experiments. Furthermore, it employs a multiprocessing concept to use all CPUs to process data efficiently in parallel. We show in our benchmark study (Tables 1 and 2) that the execution time scales almost linear with the increase of reads.

**Table 1:** Benchmark of ClinQC with Illumina Paired-end data. We used 2x100bp paired-end reads with multiple sizes ranging from 1 million to 100 million pair reads. The execution time is measured in minutes

Number of read pairs (million)	Execution time (minutes)	Read length (bp)
1	1.13	100
5	5.37	100
10	10.57	100
25	33.03	100
50	62.45	100
100	126.16	100

**Table 2:** Benchmark of ClinQC with Sanger sequencing trace files. We used 1000 trace files in AB1 format. The read lengths were ranging between 400 and 1000 base pairs. We randomly sampled 1000 files in multiple test data sets ranging from 10 files to 1000 files. The execution time is measured in minutes

Number of trace file	Execution time (minutes)
10	0.11
25	0.25
50	0.38
100	1.11
200	2.12
300	3.27
400	4.29
500	5.37
1000	11.01

### Performance evaluation

To demonstrate the performance of ClinQC we used publically available Illumina paired-end Whole Genome Sequencing (WGS) reads of CEPH/UTAH female individual (HapMap ID: NA12878) data (~420 million read pairs, 2x100bp) produced by Illumina HiSeq 2000. We downloaded seven sequencing runs from NCBI SRA (SRA ID: SRP048874). After pooling the reads from 7 libraries, we randomly sampled multiple datasets ranging in size from 1 million to 100 million. As shown in Table 1, the execution time of ClinQC time scales almost linear with the increase of read pairs.

We further evaluated the performance of ClinQC with Sanger sequencing trace files. We used 1000 trace files in AB1 format generated from the sequencing of human gene BRCA1 from 100 patients. We randomly picked trace files ranging from 10 to 1000, which could be processed in ~10 s and ~11 min, respectively. We ran all benchmarks on a Linux server (Ubuntu 12.0.4 LTS with 4 CPU, 8 GB RAM).

### ClinQC future direction

The current version of ClinQC will be extremely useful for NGS data analysis targeting whole genome sequencing, whole exome sequencing, targeted sequencing and metagenomic studies. Due to its capability to perform data analysis on Sanger data as well, it provides an integrated solution for the combined analysis of these complementary technologies. As ClinQC is an ongoing project, we will address other forthcoming quality challenges. Furthermore, we will also extend the tool to support new sequencing platforms. Currently, ClinQC is not advised for the analysis of RNA-Seq data.

### Comparison with existing tools

ClinQC provides a one-stop solution to perform various quality control steps. A comparison of the most important features of ClinQC to other available tools is given in Table 3. Many of the existing tools do not offer organization of data, parallel analysis of multiple sample/patient and none supports Sanger sequencing data analysis. Moreover, unlike other existing tools, ClinQC provides simple input options, which can be prepared as text file and allow running the whole pipeline without any manual intervention. ClinQC can be run on any operating system using a Virtual Machine, which is not offered by any other tool.

**Table 3:** Comparison of various features between ClinQC and QC tools

Features	ClinQC v1.0	CANGS v1.1	TagCleaner v0.16	SolexaQA v3.1.3	FASTX-Toolkit v0.0.13	TagDust	PRINSEQ v0.20.4	FastQC v0.11.3	NGSQCTookit v2.3.3	QC-Chain v1.0
Analysis of several datasets in a single run	yes	no	no	no	no	no	no	no	no	yes
Analysis of all platforms in single run^a^	yes	no	no	no	no	no	no	no	no	no
Virtual Machine^a^	yes	no	no	no	no	no	no	no	no	no
Sanger format conversion^a^	yes	no	no	no	no	no	no	no	no	no
Sanger base calling^a^	yes	no	no	no	no	no	no	no	no	no
Sanger QC^a^	yes	no	no	no	no	no	no	no	no	no
Sanger primer trimming^a^	yes	no	no	no	no	no	no	no	no	no
Installation required	no	yes	yes	yes	yes	yes	yes	no	no	yes
Supported sequencing platforms	Sanger, Illumina, 454, Ion torrent	454	Illumina, 454	Illumina	Illumina	Illumina, 454	Illumina, 454	any in FASTQ format	Illumina, 454	NGS
Parallel processing	yes	no	no	no	no	yes	no	yes	yes	yes
Format conversion	yes	no	no	no	yes	no	no	no	yes	yes
Primer/Adapter trimming	yes	yes	yes	no	yes	yes	no	no	yes	yes
Ns trimming	yes	yes	yes	no	no	yes	yes	no	yes	yes
Demultiplexing	yes	yes	yes	yes	yes	yes	no	no	no	yes
Detection of file format	yes	no	no	yes	no	no	yes	no	yes	yes
Dependencies	yes	yes	no	yes	yes	no	yes	no	yes	no
Graphical QC report	yes	no	no	yes	yes	no	yes	yes	yes	yes
Duplicate removal	yes	no	yes	no	yes	no	yes	no	no	yes
Contamination filtering	yes	no	yes	no	no	yes	yes	no	no	yes
GC content assessment	yes	no	yes	no	yes	no	yes	yes	yes	yes

^a^Features are unique in ClinQC

## Conclusions

ClinQC is an integrated, automated, flexible and user-friendly tool for quality control in clinical research. It supports three major NGS sequencing technologies including Illumina, 454 and Ion Torrent along with Sanger sequencing. ClinQC offers full flexibility, accuracy and reproducibility. All input parameters can be customized in the “*ClinQCOptions*” configuration file. It is a one-stop solution to run from raw sequence reads and trace files to high quality FASTQ files with Sanger quality encoding. This tool can be easily integrated in any downstream analysis pipeline for, e.g., mutation screening. In summary ClinQC can be used to analyze 1) Sanger and NGS data together, 2) all quality control parameters can be customized for different sequencing data, 3) thousands of datasets / patients / samples can be analyzed in a single run, 4) paired-end, single-end reads and mixed reads generated from Illumina, 454 and Ion Torrent can be analyzed simultaneously in a single run. ClinQC excels over existing tools and software for better usability, multiple data handling, Sanger sequencing data analysis and common input output model for Sanger and NGS data analysis.

## Availability and requirements

**Project name:** ClinQC

**Project home page:**https://sourceforge.net/projects/clinqc

**Operating system(s):** All Unix operating system

**Programming language:** Python 2.7.9

**Other requirements:** Perl 5.12 or higher, Java 1.7 or higher

**License:** LGPL

**Any restrictions to use by non-academics:** None

## Additional files

Additional file 1:
**ClinQCOptions_Sanger file to specify all input files and parameters for Sanger sequencing data analysis.** (TXT 3 kb) Additional file 2:
**ClinQCOptions_NGS file to specify all input files and parameters for Sanger sequencing data analysis.** (TXT 2 kb) Additional file 3:
**Target file (mandatory input file) to run ClinQC.** (TXT 1 kb) Additional file 4:
**Adapter-Primer file (optional input file) to perform adapters and PCR primers trimming.** (TXT 1 kb) Additional file 5:
**ClinQC user manual. An extensive guide for user to perform Sanger and NGS data analysis with ClinQC.** (PDF 1025 kb)

## Abbreviations

CPU: central processing unit
HTML: HyperText Markup Language
MACOSX: Macintosh Operating System X
MID: multiplex identifier
NCBI: National Center for Biotechnology Information
NGS: next generation sequencing
PCR: polymerase chain reaction
QC: quality control
SCF: Standard Chromatogram Format
SFF: Standard Flowgram Format
SRA: Sequence Read Archive
VM: Virtual Machine
WES: whole exome sequencing
WGS: whole genome sequencing

## References

[1] Ardeshirdavani A, Souche E, Dehaspe L, Van Houdt J, Vermeesch JR, Moreau Y (2014). NGS-Logistics: federated analysis of NGS sequence variants across multiple locations. Genome Med.

[2] Gowrisankar S, Lerner-Ellis JP, Cox S, White ET, Manion M, LeVan K (2010). Evaluation of second-generation sequencing of 19 dilated cardiomyopathy genes for clinical applications. J Mol Diagn.

[3] Valencia CA, Ankala A, Rhodenizer D, Bhide S, Littlejohn MR, Keong LM (2013). Comprehensive mutation analysis for congenital muscular dystrophy: a clinical PCR-based enrichment and next-generation sequencing panel. PLoS One.

[4] Johnston JJ, Rubinstein WS, Facio FM, Ng D, Singh LN, Teer JK (2012). Secondary variants in individuals undergoing exome sequencing: screening of 572 individuals identifies high-penetrance mutations in cancer-susceptibility genes. Am J Hum Genet.

[5] Pabinger S, Dander A, Fischer M, Snajder R, Sperk M, Efremova M (2014). A survey of tools for variant analysis of next-generation genome sequencing data. Brief Bioinform.

[6] Patel RK, Jain M (2012). NGS QC Toolkit: a toolkit for quality control of next generation sequencing data. PLoS One.

[7] FastQC. A quality control tool for high throughput sequence data. Babraham Bioinformatics Web site. http://www.bioinformatics.babraham.ac.uk/projects/fastqc/

[8] Schmieder R, Edwards R (2011). Quality control and preprocessing of metagenomic datasets. Bioinformatics.

[9] Lassmann T, Hayashizaki Y, Daub CO (2009). TagDust--a program to eliminate artifacts from next generation sequencing data. Bioinformatics.

[10] FASTX-Toolkit is a collection of command line tools for Short-Reads FASTA/FASTQ files preprocessing. Babraham Bioinformatics Web site. http://hannonlab.cshl.edu/fastx_toolkit/

[11] Cox MP, Peterson DA, Biggs PJ (2010). SolexaQA: At-a-glance quality assessment of Illumina second-generation sequencing data. BMC Bioinformatics..

[12] Schmieder R, Lim YW, Rohwer F, Edwards R (2010). TagCleaner: Identification and removal of tag sequences from genomic and metagenomic datasets. BMC Bioinformatics..

[13] Pandey RV, Nolte V, Schlötterer C (2010). CANGS: a user-friendly utility for processing and analyzing 454 GS-FLX data in biodiversity studies. BMC Res Notes..

[14] Blanca JM, Pascual L, Ziarsolo P, Nuez F, Cañizares J (2011). ngs_backbone: a pipeline for read cleaning, mapping and SNP calling using next generation sequence. BMC Genomics..

[15] Goecks J, Nekrutenko A, Taylor J, Galaxy Team (2010). Galaxy: a comprehensive approach for supporting accessible, reproducible, and transparent computational research in the life sciences. Genome Biol.

[16] Fischer M, Snajder R, Pabinger S, Dander A, Schossig A, Zschocke J (2012). SIMPLEX: cloud-enabled pipeline for the comprehensive analysis of exome sequencing data. PLoS One.

[17] Zhou Q, Su X, Wang A, Xu J, Ning K (2013). QC-Chain: fast and holistic quality control method for next-generation sequencing data. PLoS One.

[18] Criscuolo A, Brisse S (2013). AlienTrimmer: a tool to quickly and accurately trim off multiple short contaminant sequences from high-throughput sequencing reads. Genomics.

[19] TraceTuner: DNA sequencing quality values, base calling and trace processing. Sourceforge Web site. https://sourceforge.net/projects/tracetuner/.

[20] Schweyen H, Rozenberg A, Leese F (2014). Detection and removal of PCR duplicates in population genomic ddRAD studies by addition of a degenerate base region (DBR) in sequencing adapters. Biol Bull.

[21] Singh BK, Quince C, Macdonald CA, Khachane A, Thomas N, Al-Soud WA (2014). Loss of microbial diversity in soils is coincident with reductions in some specialized functions. Environ Microbiol.

[22] Dohm JC, Lottaz C, Borodina T, Himmelbauer H (2008). Substantial biases in ultra-short read data sets from high-throughput DNA sequencing. Nucleic Acids Res.

