# Supplementary material for: ClinQC: a tool for quality control and cleaning of Sanger and NGS data in clinical research
Source: BMC Bioinformatics. 2016 Feb 2;17:56. doi: 10.1186/s12859-016-0915-y (PMC4735967; doi:10.1186/s12859-016-0915-y)
Supplement: Additional file 5: — ClinQC user manual. An extensive guide for user to perform Sanger and NGS data analysis with ClinQC. (PDF 1025 kb) [file 12859_2016_915_MOESM5_ESM.pdf]

ClinQC User Guide, Version 1.0

Health & Environment Department
AIT Austrian Institute of Technology GmbH
Vienna 1190, Austria
December 08 2015

TABLE OF CONTENTS

1. What is ClinQC? …………………………………………………………….. 3

2. System requirements ………………………………………………………... 4

3. Software/tools required ……………………………………………………... 4

4. How to obtain ClinQC? ……………………………………………………… 4

    4.1 Download ClinQC Virtual Machine …………………………………. 4

        4.1.1 How to use ClinQC Virtual Machine? …………………….. 5

    4.2 Download ClinQC source code ……………………………..………. 5

    4.3 Run ClinQC with test inputs ………………………………………….. 6

5. ClinQC inputs requirements ………………………………………………..... 7

6. How to use ClinQC? ………………………………………………………… 7

    6.1 How to analyze Sanger data?..……………………………………… 8

    6.2 How to analyze NGS data?...………………………………………... 9

7. ClinQC outputs description ………………………………………………... 12

8. Contact information ………………………………………………………… 13

1. What is ClinQC?

ClinQC is an integrated pipeline for quality control, filtering and trimming of Sanger and NGS sequencing data for hundred to thousands of samples/patients in a single run in clinical research. It can analyze Sanger sequencing and NGS data from raw reads and produces unified output as FASTQ files per sample/patient with Sanger quality encoding. The important features of ClinQC are described below:

1. ClinQC supports three major NGS platforms including Illumina, 454/Roche and Ion torrent.
2. ClinQC supports Sanger sequencing data analysis from trace file to FASTQ file with Sanger quality encoding
3. ClinQC supports Single-end and Paired-end reads.
4. ClinQC can be used to analyze the sequencing data generated from single-gene-panel, multigene-panel, exome-seq, genome-seq and RNA-seq experiments.
5. ClinQC has uniform input and output model for Sanger and NGS data analysis.
6. ClinQC can be used to analyze several patients/samples in a single run simultaneously.
7. ClinQC can be used to analyze Sanger and NGS sequencing data simultaneously in a single run.

2. System requirements

Operating system

Linux

Mac OSX 10.6 or later

Windows PC

Software

Python 2.7.9

Biopython 1.60 or higher

Bioperl 1.6 or higher

Perl 5.10 or higher

Java 1.7 or higher

3. Software/tools required

AlienTrimmer [ftp://ftp.pasteur.fr/pub/gensoft/projects/AlienTrimmer/]

TraceTuner [https://sourceforge.net/projects/tracetuner/]

FASTQC [http://www.bioinformatics.babraham.ac.uk/projects/fastqc/]

PRINSEQ [http://sourceforge.net/projects/prinseq/files/standalone/]

4. How to obtain ClinQC?

We provide source code in two version of ClinQC for Linux, and Macintosh operating systems. For Windows PC users we provide a fully configured Virtual Machine (VM can be used on any operating system). Along with source code and virtual machine we provide test data and extensive user manual for step-by-step get and run ClinQC for expert and non-expert users.

4.1 Download ClinQC Virtual Machine

For all users we provide a fully configured Virtual Machine (VM), which is

readily available and thus does not require any installation and configuration and works on any operating system including Windows, Linux and Mac osx. The VM can be obtained from https://sourceforge.net/p/clinqc/wiki/Virtual_Machine/

4.1.1 How to use ClinQC Virtual Machine?

Step1: Download and install Virtual Box from https://www.virtualbox.org/.

Step2: Download ClinQC Virtual machine from https://sourceforge.net/p/clinqc/wiki/Virtual_Machine

Step3: Import ClinQC Virtual Machine file into Virtual Box.

Step4: Login into ClinQC Virtual machine with username and password = **testuser**

Step5: Open terminal and change to ***ClinQC_v1.0*** directory with following command:

```
cd ~/ClinQC_v1.0
```

Step6: Run ClinQC pipeline with following command

```
sh ./run_clinqc.sh
```

Step7: To run ClinQC pipeline with other data analysis, prepare the Target file and ClinQCOption file to run the ClinQC pipeline.

4.2 Download ClinQC source code

If user wants to use ClinQC on own system then user can download the latest version of ClinQC source code (1) for Linux computers from https://sourceforge.net/projects/clinqc/files/ClinQC_v1.0-linux.zip and 2) for Macintosh OSX computers form

https://sourceforge.net/projects/clinqc/files/ClinQC_v1.0-macos.zip. Move the file to an appropriate directory and run the following command to uncompress the file:

unzip ClinQC_v1.0-linux.zip

For Macintosh version run following command to unzip ClinQC source code

unzip ClinQC_v1.0-macos.zip

Note that after uncompressing the **.zip** file, a new folder will be created named ***ClinQC_v1.0***. This directory contains the following files and folders. Files are denoted in blue and sub folders are denoted in red colors:

< ClinQC_v1.0>

|

- < clinqc>
- <ClinQCOptions_Sanger>
- <ClinQCOptions_NGS>
- <bin>
- <executables>

  |

  - <AlienTrimmer>
  - <FastQC>
  - <TraceTuner>
  - <PRINSEQ>

4.3 Run ClinQC with test input

To validate the installation of the ClinQC pipeline it can be run with a small test data set. The test data set and the corresponding ClinQC configuration files for Sanger and NGS can be obtained from https://sourceforge.net/projects/clinqc/files/test_data.zip and download in ClinQC_v1.0 folder/directory and run the following command to uncompress the file:

unzip test_data.zip

Note that after uncompressing the **.zip** file, a new folder will be created named

test_data in ClinQC_v1.0 folder. And then run following two commands to run Sanger data analysis and NGS data analysis.

Sanger analysis:

cd ~/ClinQC_v1.0

./clinqc --option_file ClinQCOptions_Sanger

NGS analysis:

cd ~/ClinQC_v1.0

./clinqc_v1.0 –option ClinQCOptions_NGS

To get help on how to run ClinQC and required parameters enter:

./*clinqc –help*

5. ClinQC inputs requirements

ClinQC pipeline has several sequential steps that can be run by a single command. All input parameters can be specified in the **ClinQCOptions file**. These parameters are then used to run the whole pipeline. ClinQC provides two different input options file for Sanger and NGS:

ClinQCOptions_Sanger: This configuration file can be used for Sanger sequencing data analysis for quality control, trimming and filtering to obtain high quality FASTQ files. We have already given default value for required parameters to run the whole Sanger sequencing analysis from raw reads to high FASTQ file.

ClinQCOptions_NGS: This configuration file can be used for NGS sequencing data analysis for quality control, trimming and filtering to obtain high quality FASTQ files. We have already given default value for required parameters to run the whole Sanger sequencing analysis from raw reads to high FASTQ file.

ClinQC can be run with the following command, which should be run under the folder/directory *ClinQC_v1.0* directory:

./*clinqc --option-file <path to ClinQCOptions file>*

However, before running ClinQC with your own dataset all parameters have to be specified in the appropriate ClinQC Options files (*ClinQCOptions_Sanger* OR *ClinQCOptions_NGS*).

6. How to use ClinQC?

To use ClinQC for quality control, trimming and filtering Sanger sequencing files should be in AB1 and SCF format, Illumina reads in FASTQ (both paired end and single end), 454 reads in SFF and FASTA-QUAL and Ion torrent reads in SFF and FASTQ format.

6.1 How to analyze Sanger data?

Step1: Prepare Input files:

1. Prepare Target file

For each analysis user need to prepare a target file in a predefined format. It is a *tab-separated* text file, which contains 10 columns. As shown in figure 1, one row for each sequencing file in target file. Target file is a mandatory input, which must be provided. The target file can be given in the ClinQCOptions file with the input name **Target_File="sanger_target_file.txt"**

Patient_Id	Family_Id	Assay_Id	Lab_Analysis_Date	Platform	Seq_System	Read_Type	File_Path	File_Format	Barcode
OO230.2	OO230	ASSanger1	2014-01-27_15-55-12	Sanger	ABI Seq	Single-End	test_data/sanger/ab1/OO230_2/1866_001_1_OO230_2_BR1EX9_F_O01.ab1	ab1	
OO230.2	OO230	ASSanger1	2014-01-27_15-55-13	Sanger	ABI Seq	Single-End	test_data/sanger/ab1/OO230_2/1866_002_1_OO230_2_BR1EX8_F_M01.ab1	ab1	
OO230.2	OO230	ASSanger1	2014-01-27_15-55-13	Sanger	ABI Seq	Single-End	test_data/sanger/ab1/OO230_2/1866_003_1_OO230_2_BR1EX7_2_F_K01.ab1	ab1	
OO230.2	OO230	ASSanger1	2014-01-27_15-55-13	Sanger	ABI Seq	Single-End	test_data/sanger/ab1/OO230_2/1866_004_1_OO230_2_BR1EX7_1_F_I01.ab1	ab1	
OO230.2	OO230	ASSanger1	2014-01-27_15-55-13	Sanger	ABI Seq	Single-End	test_data/sanger/ab1/OO230_2/1866_005_1_OO230_2_BR1EX6_F_G01.ab1	ab1	
OO230.2	OO230	ASSanger1	2014-01-27_15-55-13	Sanger	ABI Seq	Single-End	test_data/sanger/ab1/OO230_2/1866_006_1_OO230_2_BR1EX5_F_E01.ab1	ab1	
OO230.2	OO230	ASSanger1	2014-01-27_15-55-13	Sanger	ABI Seq	Single-End	test_data/sanger/ab1/OO230_2/1866_007_1_OO230_2_BR1EX3_F_C01.ab1	ab1	
OO230.2	OO230	ASSanger1	2014-01-27_15-55-13	Sanger	ABI Seq	Single-End	test_data/sanger/ab1/OO230_2/1866_008_1_OO230_2_BR1EX2_F_A01.ab1	ab1	
OO230.2	OO230	ASSanger1	2014-01-27_15-55-01	Sanger	ABI Seq	Single-End	test_data/sanger/ab1/OO230_2/1866_009_1_OO230_2_BR1EX14_F_O05.ab1	ab1	
OO230.2	OO230	ASSanger1	2014-01-27_15-55-01	Sanger	ABI Seq	Single-End	test_data/sanger/ab1/OO230_2/1866_010_1_OO230_2_BR1EX13_F_M05.ab1	ab1	
OO230.2	OO230	ASSanger1	2014-01-27_15-55-01	Sanger	ABI Seq	Single-End	test_data/sanger/ab1/OO230_2/1866_011_1_OO230_2_BR1EX12_F_K05.ab1	ab1	
OO230.2	OO230	ASSanger1	2014-01-27_15-55-01	Sanger	ABI Seq	Single-End	test_data/sanger/ab1/OO230_2/1866_012_1_OO230_2_BR1EX11l_F_I05.ab1	ab1	
OO230.2	OO230	ASSanger1	2014-01-27_15-55-01	Sanger	ABI Seq	Single-End	test_data/sanger/ab1/OO230_2/1866_013_1_OO230_2_BR1EX11k_F_G05.ab1	ab1	
OO230.2	OO230	ASSanger1	2014-01-27_15-55-02	Sanger	ABI Seq	Single-End	test_data/sanger/ab1/OO230_2/1866_014_1_OO230_2_BR1EX11j_F_E05.ab1	ab1	
OO230.2	OO230	ASSanger1	2014-01-27_15-55-02	Sanger	ABI Seq	Single-End	test_data/sanger/ab1/OO230_2/1866_015_1_OO230_2_BR1EX11i_F_C05.ab1	ab1	
OO230.2	OO230	ASSanger1	2014-01-27_15-55-02	Sanger	ABI Seq	Single-End	test_data/sanger/ab1/OO230_2/1866_016_1_OO230_2_BR1EX11h_F_A05.ab1	ab1	
OO230.2	OO230	ASSanger1	2014-01-27_15-55-02	Sanger	ABI Seq	Single-End	test_data/sanger/ab1/OO230_2/1866_017_1_OO230_2_BR2EX6_7_F_O09.ab1	ab1	
OO230.2	OO230	ASSanger1	2014-01-27_15-55-02	Sanger	ABI Seq	Single-End	test_data/sanger/ab1/OO230_2/1866_018_1_OO230_2_BR2EX5_6_F_M09.ab1	ab1	
OO230.2	OO230	ASSanger1	2014-01-27_15-55-02	Sanger	ABI Seq	Single-End	test_data/sanger/ab1/OO230_2/1866_019_1_OO230_2_BR2EX4_F_K09.ab1	ab1	
OO230.2	OO230	ASSanger1	2014-01-27_15-55-02	Sanger	ABI Seq	Single-End	test_data/sanger/ab1/OO230_2/1866_020_1_OO230_2_BR2EX3_2_F_I09.ab1	ab1	
OO230.2	OO230	ASSanger1	2014-01-27_15-55-03	Sanger	ABI Seq	Single-End	test_data/sanger/ab1/OO230_2/1866_021_1_OO230_2_BR2EX3_1_F_G09.ab1	ab1	
OO230.2	OO230	ASSanger1	2014-01-27_15-55-03	Sanger	ABI Seq	Single-End	test_data/sanger/ab1/OO230_2/1866_022_1_OO230_2_BR2EX2_F_E09.ab1	ab1	
OO230.2	OO230	ASSanger1	2014-01-27_15-55-03	Sanger	ABI Seq	Single-End	test_data/sanger/ab1/OO230_2/1866_023_1_OO230_2_BR1EX24_F_C09.ab1	ab1	
OO230.2	OO230	ASSanger1	2014-01-27_15-55-03	Sanger	ABI Seq	Single-End	test_data/sanger/ab1/OO230_2/1866_024_1_OO230_2_BR1EX23_F_A09.ab1	ab1	

Figure 1: Shows Target file for Sanger data analysis

2. Prepare ClinQCOptions_Sanger file

ClinQC requires an input configuration file, which can be prepared once by customizing parameters as per the requirement, and the whole pipeline will be run without further user interaction.

(1) Set the **Output file and directory location** as shown in Figure 2 below

```
##############################################################################################################################
#
#                                       Output file and directory location
#
##############################################################################################################################

### Provide the output directory
Output_Dir = "test_output/sanger_output_dir"

### Provide the final output file name
Output_File = "test_output/sanger_output_file"
```

Figure 2: Set Output director and output file name and path

(2) Set the **Quality control and filtering** as shown in Figure 3 below

```
##############################################################################################################################
#
###             Step1: Quality control and filtering
#
##############################################################################################################################

### Minimum average base quality for trimming the low quality 5' and 3' end of reads
Minimum_Base_Quality = 20

### Minimum read length to discard the read for further analysis
Minimum_Read_Length = 50

### Maximum read length to discard the read for further analysis
Maximum_Read_Length = 1000

### Sanger data quality control and trimming parameters for TTUNER
Sanger_Trim_Window_Size = 10
Sanger_Trim_Base_Quality = 20
```

Figure 3: Set Quality control and filtering parameters

(3) Set the **Third party software/tool executable path** as shown in Figure 4 below

```
##############################################################################################################################
#
# Third party software/tool executables
# Provide the full path to the third party executables to run the ClinQC pipeline.
#
#
##############################################################################################################################


# Provide the full path of the executables relative to the <ClinQC_1.0>

###### FASTQC executables
FASTQC = executables/FastQC/fastqc

###### Sanger ab1 and scf file QC tool
TTUNER = executables/tracetuner_3.0.6beta/rel/Linux_64/ttuner

###### AlienTrimmer jar file for primers and adapter trimming
AlienTrimmer = executables/AlienTrimmer_0.4.0/src/AlienTrimmer.jar
```

Figure 4: Specifications of full paths of external software/tools used in

ClinQC.

Step2: Run ClinQC pipeline:

After preparing the Target File and ClinQCOptions file have been prepared and customized then ClinQC pipeline can be run with following command line

./clinqc –option_file ClinQCOptions_Sanger

6.2 How to analyze NGS data?

Step1: Prepare Input files:

1. Prepare Target file

For each analysis user need to prepare a target file in a predefined format. It is a *tab-separated* text file, which contains 10 columns. As shown in below figure 5, one row for each sequencing file in target file. Target file is a mandatory input, which must be provided. The target file can be given in the ClinQCOptions file with the input name **Target_File="ngs_target_file.txt"**

(a)

Patient_Id	Family_Id	Assay_Id	Lab_Analysis_Date	Platform	Seq_System	Read_Type	File_Path	File_Format	Barcode
p000001	F001	BRCA1_Illumina	2013-10-30_18-42-59	Illumina	HiSeq	Paired-End	test_data/illumina/SRR1611183_chr1_1_1M.fastq;test_data/illumina/SRR1611183_chr1_2_1M.fastq	fastq;fastq	
p000002	F001	BRCA1_Illumina	2013-10-30_18-42-59	Illumina	HiSeq	Paired-End	test_data/illumina/SRR1611183_chr1_1_5M.fastq;test_data/illumina/SRR1611183_chr1_2_5M.fastq	fastq;fastq	
p000003	F001	BRCA1_Illumina	2013-10-30_18-42-59	Illumina	HiSeq	Single-End	test_data/illumina/SRR1611183_chr1_1_1MS.fastq	fastq	
p000004	F001	BRCA1_Illumina	2013-10-30_18-42-59	Illumina	HiSeq	Single-End	test_data/illumina/SRR1611183_chr1_1_5MS.fastq	fastq	

(b)

Patient_Id	Family_Id	Assay_Id	Lab_Analysis_Date	Platform	Seq_System	Read_Type	File_Path	File_Format	Barcode
p000001	F001	AS454	2014-02-06_14-16-39	Roche	GS Junior	Single-End	test_data/454/fasta_qual/test1.fna;data/test_data/454/fasta_qual/test1.qual	fasta;qual	ACGAGTGCGT
p000002	F001	AS454	2014-02-06_14-16-39	Roche	GS Junior	Single-End	test_data/454/fasta_qual/test1.fna;data/test_data/454/fasta_qual/test1.qual	fasta;qual	ACGCTCGACA
p000003	F001	AS454	2014-02-06_14-16-39	Roche	GS Junior	Single-End	test_data/454/fasta_qual/test1.fna;data/test_data/454/fasta_qual/test1.qual	fasta;qual	AGACGCACTC
p000004	F001	AS454	2014-02-06_14-16-39	Roche	GS Junior	Single-End	test_data/454/fasta_qual/test1.fna;data/test_data/454/fasta_qual/test1.qual	fasta;qual	AGCACTGTAG
p000005	F001	AS454	2014-02-06_14-16-39	Roche	GS Junior	Single-End	test_data/454/fasta_qual/test1.fna;data/test_data/454/fasta_qual/test1.qual	fasta;qual	ATCAGACACG
p000006	F001	AS454	2014-02-06_14-16-39	Roche	GS Junior	Single-End	test_data/454/fasta_qual/test1.fna;data/test_data/454/fasta_qual/test1.qual	fasta;qual	ATATCGCGAG

(c)

Patient_Id	Family_Id	Assay_Id	Lab_Analysis_Date	Platform	Seq_System	Read_Type	File_Path	File_Format	Barcode
p000001	F001	ASPGM	2014-02-06_14-16-39	iontorrent	PGM	Single-End	test_data/iontorrent/sff/IOE5T0W01.sff	sff	ACGAGTGCGT
p000002	F001	ASPGM	2014-02-06_14-16-39	iontorrent	PGM	Single-End	test_data/iontorrent/sff/IOE5T0W01.sff	sff	ACGCTCGACA
p000003	F001	ASPGM	2014-02-06_14-16-39	iontorrent	PGM	Single-End	test_data/iontorrent/sff/IOE5T0W01.sff	sff	AGACGCACTC
p000004	F001	ASPGM	2014-02-06_14-16-39	iontorrent	PGM	Single-End	test_data/iontorrent/sff/IOE5T0W01.sff	sff	AGCACTGTAG
p000005	F001	ASPGM	2014-02-06_14-16-39	iontorrent	PGM	Single-End	test_data/iontorrent/sff/IOE5T0W01.sff	sff	ATCAGACACG
p000006	F001	ASPGM	2014-02-06_14-16-39	iontorrent	PGM	Single-End	test_data/iontorrent/sff/IOE5T0W01.sff	sff	ATATCGCGAG

Figure 5: Shows different Target files for NGS data analysis. (a) Illumina analysis, (b) 454 analysis, and (c) Ion torrent

2. Prepare ClinQCOptions_NGS file

ClinQC requires an input configuration file, which can be prepared once by customizing parameters as per the requirement, and the whole pipeline will be run without further user interaction.

(1) Set the **Output file and directory location** as shown in Figure 6 below

```
##########################################################################################################################
#
#                                    Output file and directory location
#
##########################################################################################################################

### Provide the output directory
Output_Dir = "test_output/454_output_dir"

### Provide the final output file name
Output_File = "test_output/454_output_file"
```

Figure 6: Set Output director and output file name and path

(2) Set the **Quality control and filtering** as shown in Figure 7 below

```
##########################################################################################################################
#
###             Step1: Quality control and filtering
#
##########################################################################################################################

### Minimum average base quality for trimming the low quality 5' and 3' end of reads
Minimum_Base_Quality = 20

### Minimum read length to discard the read for further analysis
Minimum_Read_Length = 50

### Maximum read length to discard the read for further analysis
Maximum_Read_Length = 1000
```

Figure 7: Set Quality control and filtering parameters

(3) Set the **Third party software/tool executable path** as shown in Figure 8 below

```
##########################################################################################################################
#
# Third party software/tool executables
# Provide the full path to the third party executables to run the ClinQC pipeline.
#
#
##########################################################################################################################


# Provide the full path of the executables relative to the <ClinQC_1.0>

###### FASTQC executables
FASTQC = executables/FastQC/fastqc

###### Sanger ab1 and scf file QC tool
TTUNER = executables/tracetuner_3.0.6beta/rel/Linux_64/ttuner

###### AlienTrimmer jar file for primers and adapter trimming
AlienTrimmer = executables/AlienTrimmer_0.4.0/src/AlienTrimmer.jar
```

Figure 8: Specifications of full paths of external software/tools used in ClinQC.

Step2: Run ClinQC pipeline:

After preparing the Target File and ClinQCOptions file have been prepared and customized then ClinQC pipeline can be run with following command line

./clinqc –option_file ClinQCOptions_NGS

7. ClinQC outputs description

After running ClinQC, the results of the ClinQC pipeline can be found in the output directory (as specified in the ClinQC Options file). Results are provided in the following format.

```
<OUTPUT_FILE>
<OUTPUT_DIR>
        |
        - <fastq_files>
                |
                - <patient1_1.fq;patient1_2.fq>
                - <patient2_1.fq;patient2_2.fq>
                - <patient3_1.fq;patient3_2.fq>
                - .
                - .
                - .
                - <patientN_1.fq;patientN_2.fq>

        - <QC_report>
                |
                - <before_qc_patient1_1.fq_fastqc_qc_report.html>
                - <after_qc_patient1_1.fq_fastqc_qc_report.html>
                - <before_qc_patient1_2.fq_fastqc_qc_report.html>
                - <after_qc_patient1_2.fq_fastqc_qc_report.html>
                - .
                - .
                - .     |
                - .
                - <before_qc_patientN_1.fq_fastqc_qc_report.html>
                - <after_qc_patientN_1.fq_fastqc_qc_report.html>
                - <before_qc_patientN_2.fq_fastqc_qc_report.html>
                - <after_qc_patientN_2.fq_fastqc_qc_report.html>
```

<fastq_files>:

This output folder contains high quality FASTQ file for each patient with Sanger quality encoding. If reads are in paired-end then there will be two files for each patient.

<QC_report>:

This output folder contains Quality control and trimming report in html format generated by FASTQC tool. There are two files for each FASTQ files 1) before quality control and 2) after quality control.

8. Contact Information

PD Dr. Andreas Weinhäusel
andreas.weinhaeusel@ait.ac.at
Dr. Albert Kriegner
albert.kriegner@platomics.com
Ram Vinay Pandey
ramvinay.pandey@gmail.com
